# Supplementary material for: Electromechanical coupling mechanism for activation and inactivation of an HCN channel
Source: Nat Commun. 2021 May 14;12:2802. doi: 10.1038/s41467-021-23062-7 (PMC8121817; doi:10.1038/s41467-021-23062-7)
Supplement: Supplementary file 1 — Supplementary Information [file 41467_2021_23062_MOESM1_ESM.pdf]

Electromechanical Coupling Mechanism for Activation and Inactivation of an HCN channel

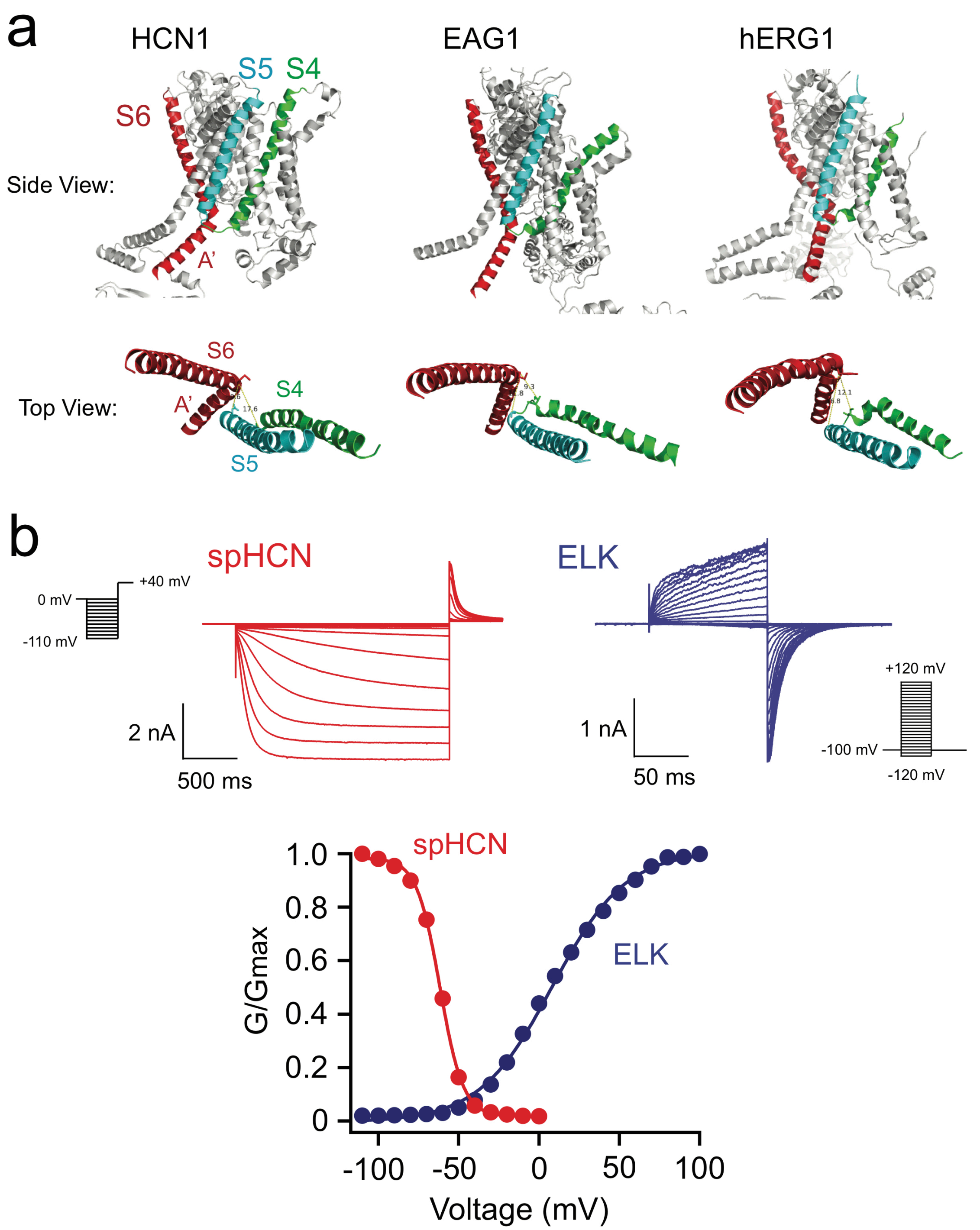

Supplementary Figure 1

Hyperpolarization-activated versus depolarization-activated CNBD-family channels.

a, Side view and top view of the cryo-EM structures of HCN1 (PDB: 5U6O), EAG1 (PDB: 5K7L), and hERG1 (PDB: 5VA2), highlighting the similar arrangement of S4, S5 and S6/A' helices. **b**, Exemplar current recordings of zebra fish ELK (KCNH)[1] and spHCN channels and their reversed conductance-voltage (G-V) relationships.

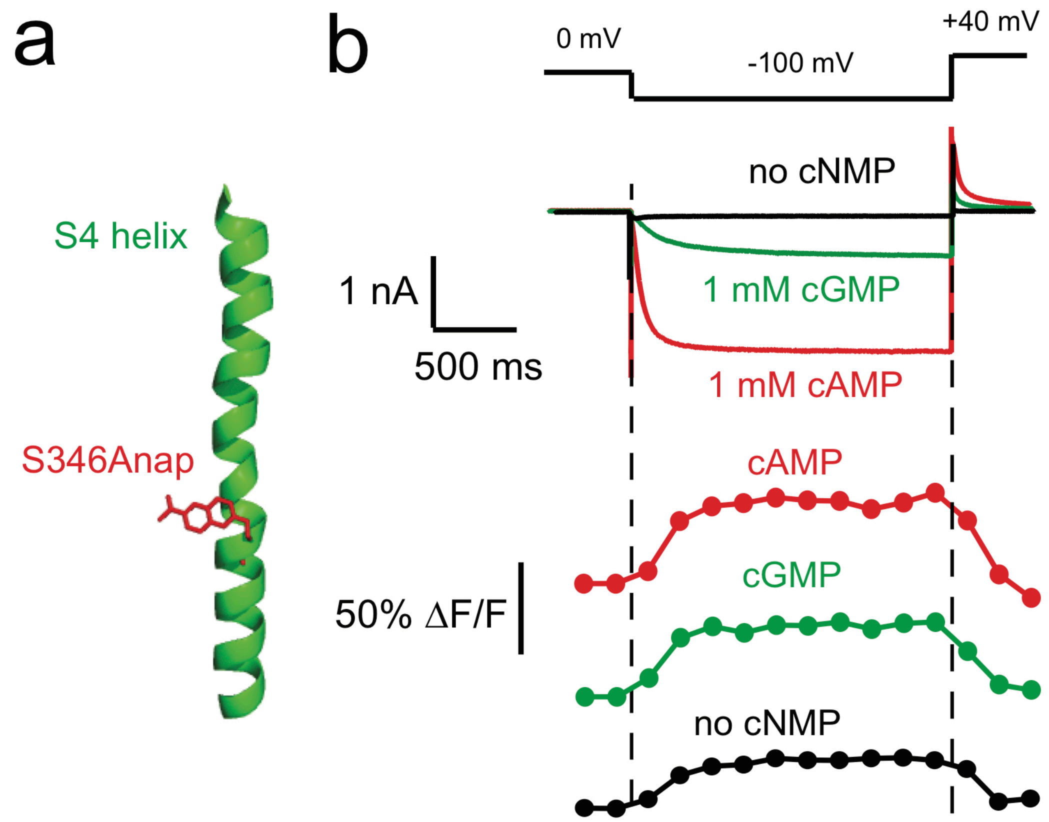

Supplementary Figure 2

Detecting the VSD movement of spHCN channels using the environmental sensitivity of L-Anap incorporated in the Ser^{346} position of the S4 helix.

a, Illustration of the incorporation of L-Anap in the Ser^{346} position of the S4 helix. **b**, Simultaneous current (top) and fluorescence (bottom) measurements from spHCN-S346Anap channels in response to a -100 mV hyperpolarizing pulse in 1 mM cAMP (red), 1 mM cGMP (green) or in the absence of cyclic nucleotide (black).

Electromechanical Coupling Mechanism for Activation and Inactivation of an HCN channel

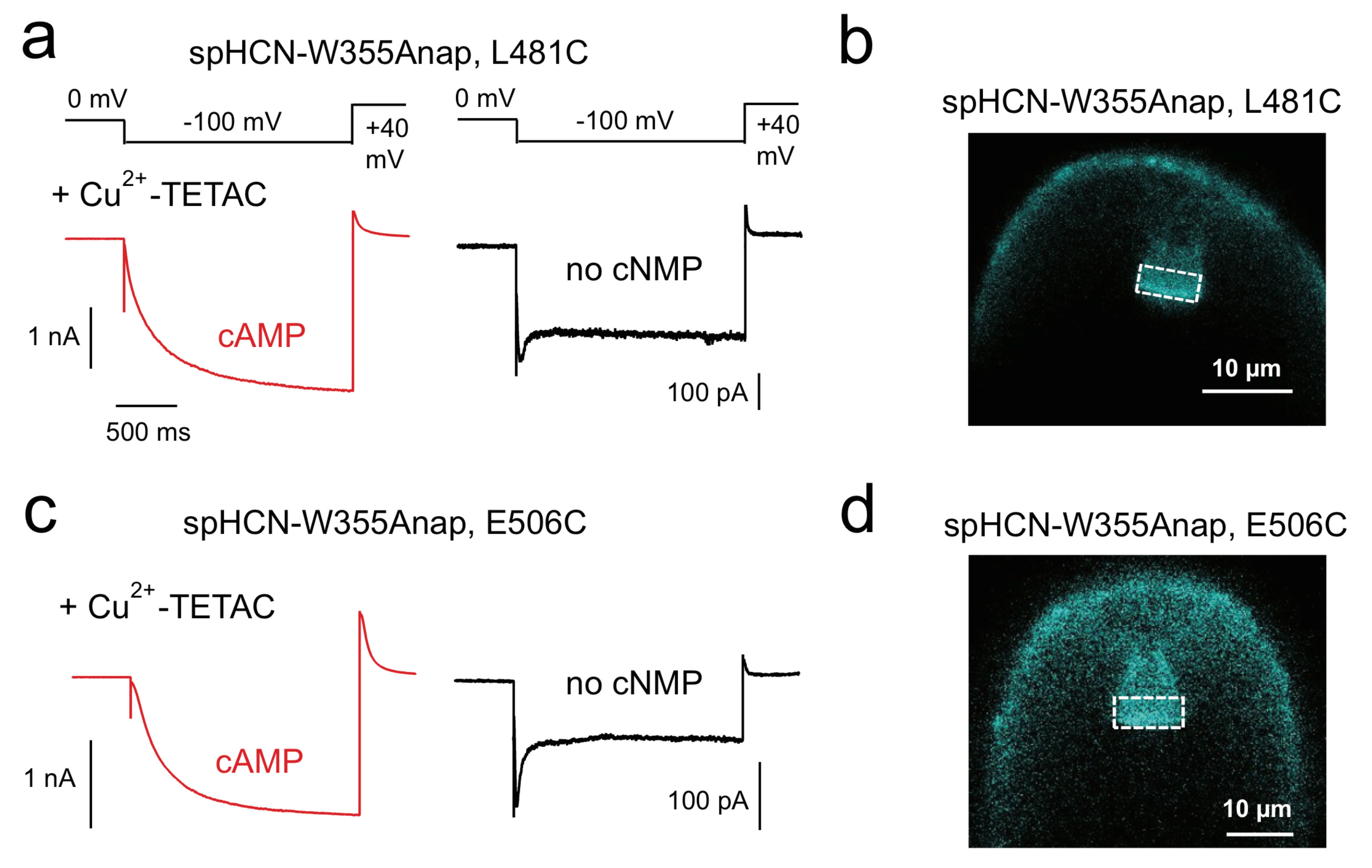

Supplementary Figure 3

Functional and fluorescence measurements of spHCN-W355Anap channels.

a, Representative current recordings of spHCN-W355Anap, L481C channels in response to a -100 mV hyperpolarizing pulse in 1 mM cAMP or in the absence of cyclic nucleotide after applying Cu^{2+}-TETAC. **b**, Exemplar PCF images of spHCN-W355Anap, L481C channels. The dashed square indicates the area of the patch membrane for quantifying Anap fluorescence intensities. **c**, Representative current recordings of spHCN-W355Anap, E506C channels in response to a -100 mV hyperpolarizing pulse in 1 mM cAMP or in the absence of cyclic nucleotide after applying Cu^{2+}-TETAC. **d**, Exemplar PCF images of spHCN-W355Anap, L481C channels.

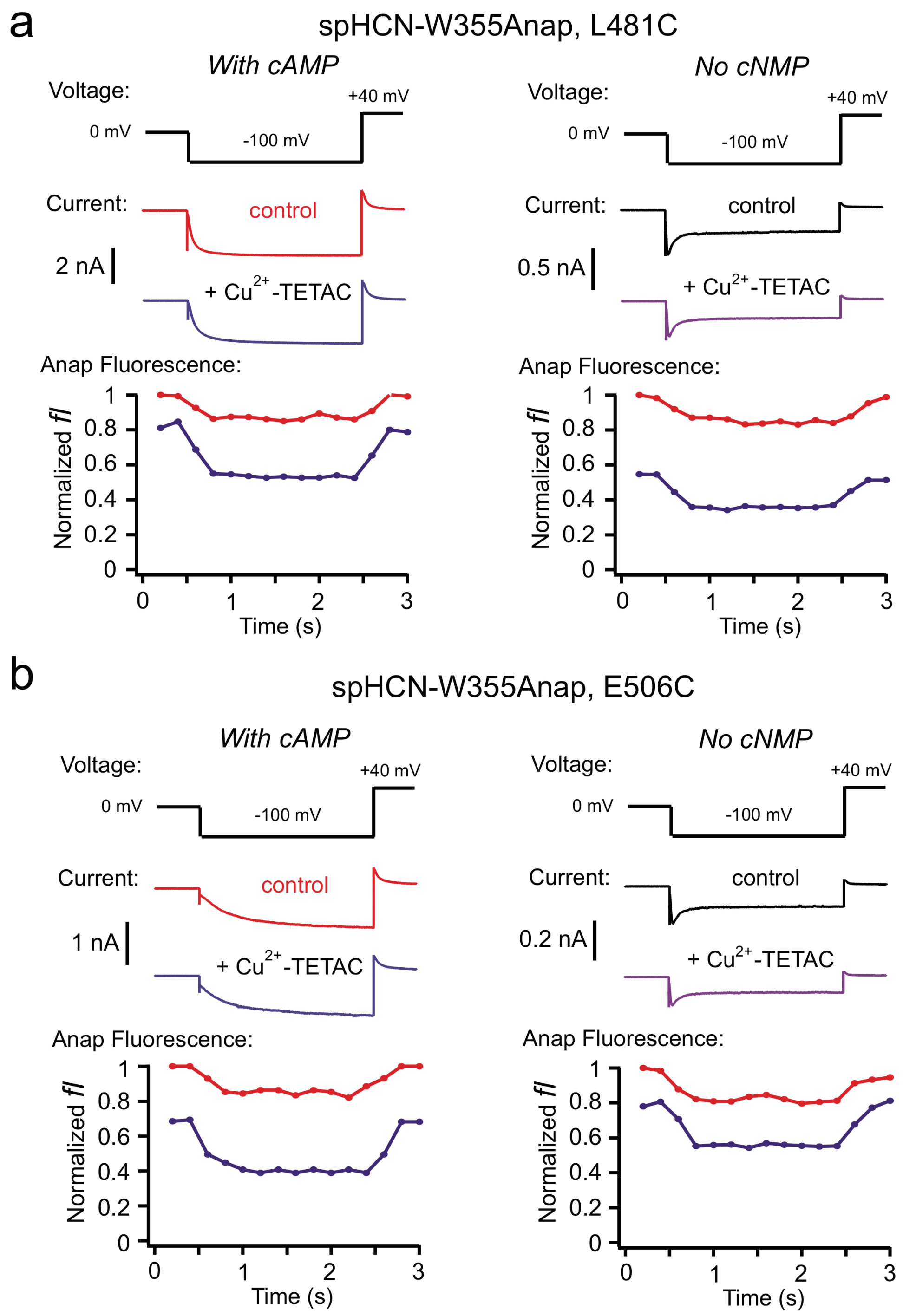

Supplementary Figure 4

tmFRET experiments with simultaneous current and fluorescence measurements for spHCN-W355Anap channels.

a, Simultaneous current (top) and fluorescence (bottom) measurements from spHCN-W355Anap, L481C channels, in the presence of 1 mM cAMP (left) or in the absence of cyclic nucleotide (right), before (red or black) and after (purple) applying Cu^{2+}-TETAC. **b**, Simultaneous current (top) and fluorescence (bottom) measurements from spHCN-W355Anap, E506C channels, in the presence of 1 mM cAMP (left) or in the absence of cyclic nucleotide (right), before (red or black) and after (purple) applying Cu^{2+}-TETAC.

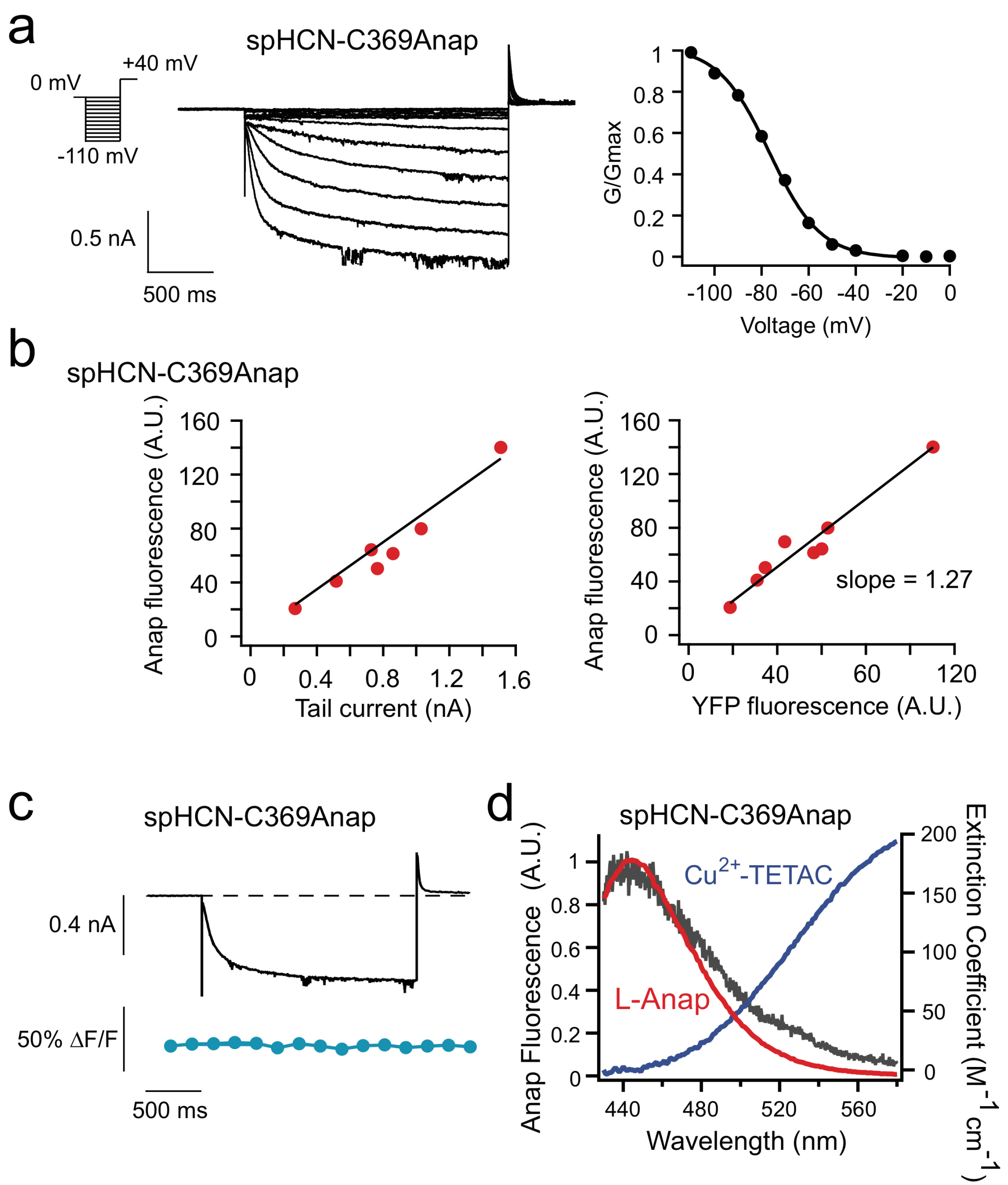

Supplementary Figure 5

Functional and fluorescence properties of spHCN-C369Anap channels.

a, Representative current recordings and G-V relationship of spHCN-C369Anap channels in 1 mM cAMP. **b**, Correlations of Anap fluorescence intensity with ionic current amplitude (left) and YFP fluorescence intensity (right) for spHCN-C369Anap channels. **c**, Simultaneous current (top) and fluorescence (bottom) measurements from spHCN-C369Anap channels in response to a -100 mV hyperpolarizing pulse in 1 mM cAMP. Similar experiment for spHCN-W355Anap channels was reported in a previous paper[2]. **d**, Spectral properties of L-Anap emission of spHCN-C369Anap channels and transition metal ion absorption by Cu^{2+}-TETAC. Red trace is the corrected spectra used for the R_0 calculation from known spectra of free L-Anap as described previously[2].

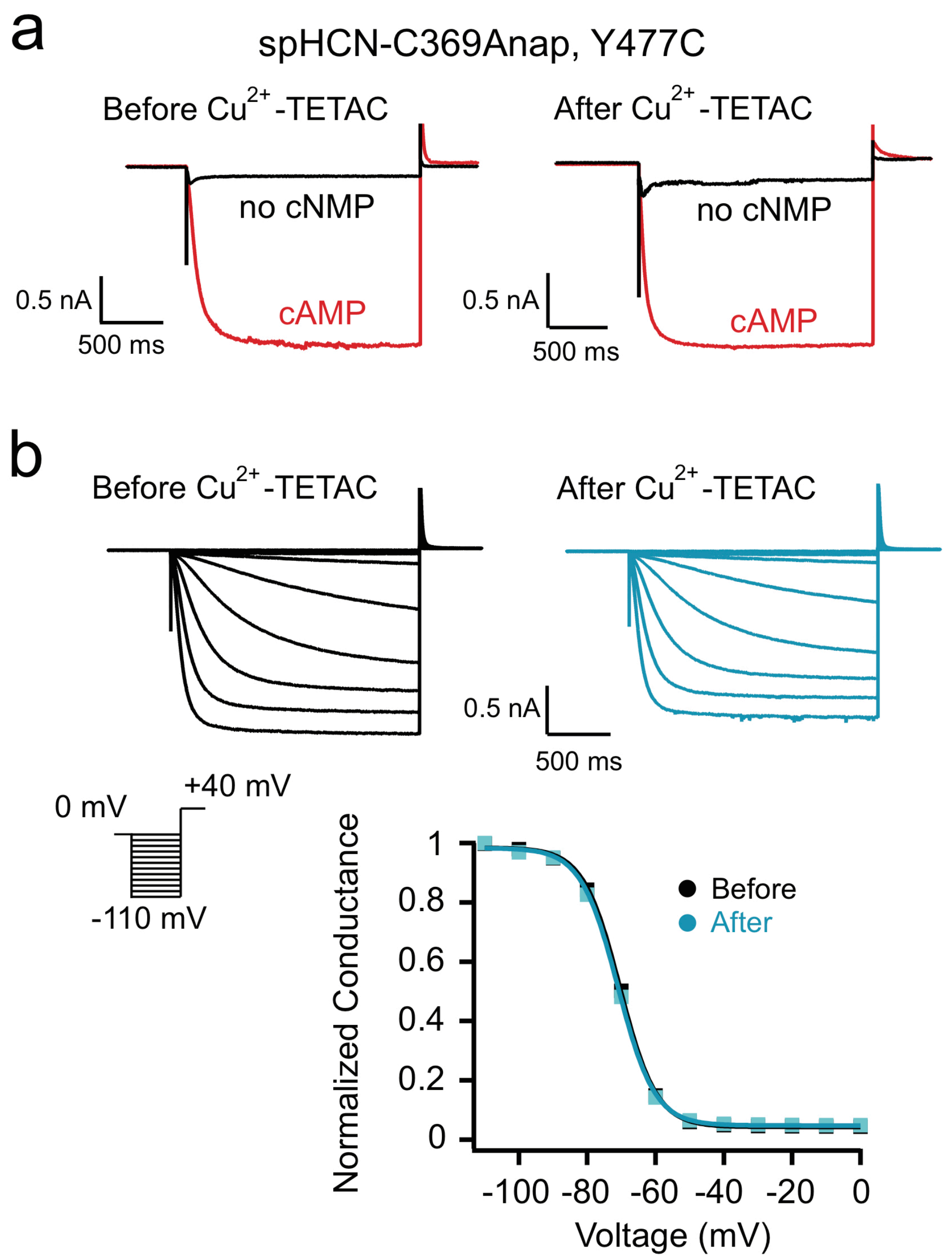

Supplementary Figure 6

Effects of Cu^{2+}-TETAC on spHCN-C369Anap, Y477C channels.

a, Representative current recordings of spHCN-C369Anap, Y477C channels in the presence of cAMP or in the absence of cyclic nucleotide (no cNMP) before (left) and after (right) applying Cu^{2+}-TETAC. Voltage protocol is the same as in Fig S3 and Fig S4. **b**, Currents elicited by steps of hyperpolarizing voltages and the G-V relationships of spHCN-C369Anap, Y477C channels in the presence of 1 mM cAMP before and after applying Cu^{2+}-TETAC.

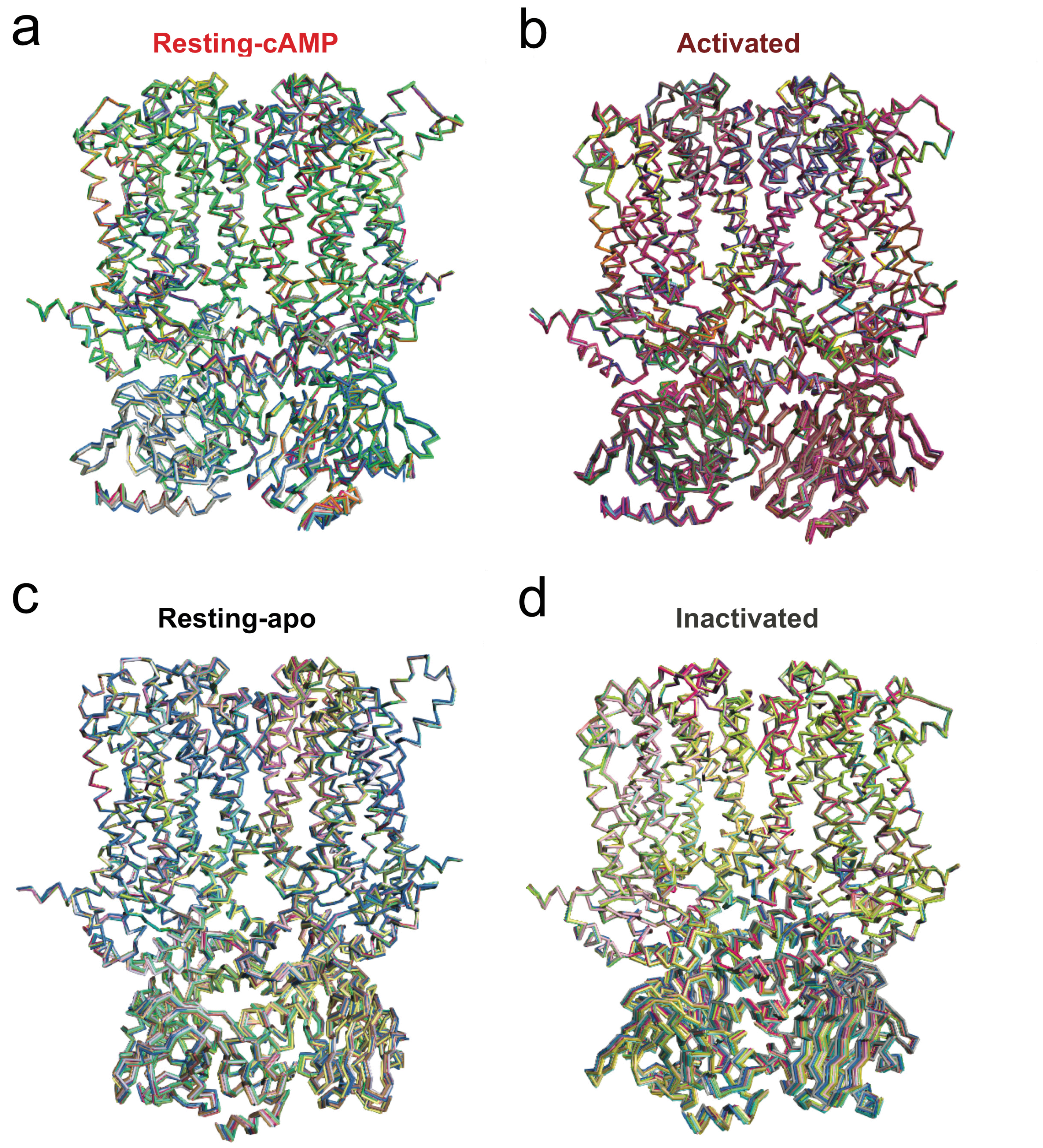

Supplementary Figure 7

Convergence of Rosetta models

a-d, Overlays of the top 17 lowest energy models for each state as in Fig 6, showing the high convergence of the models. The Cα RMSD of these models is less than 1 Å.

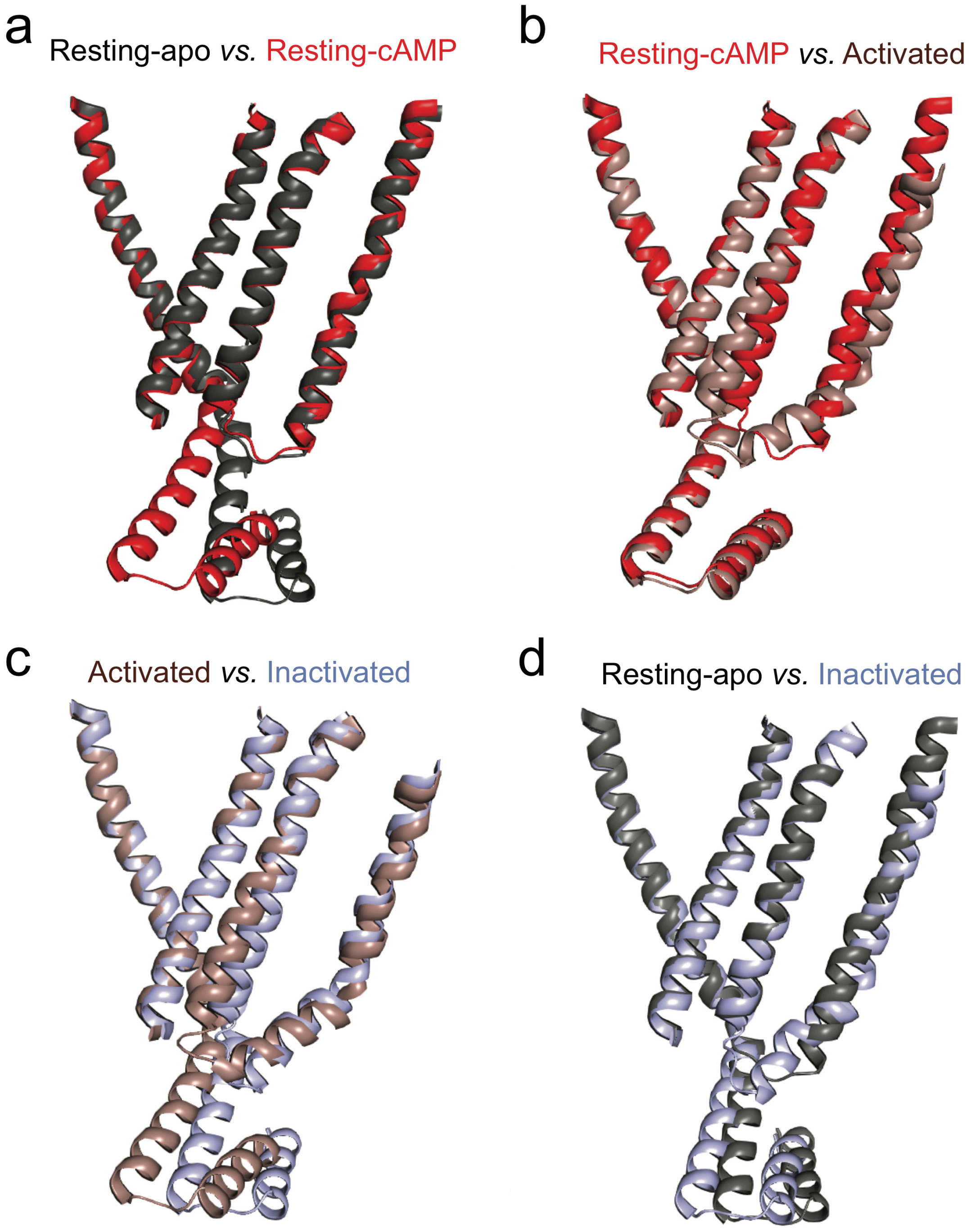

Supplementary Figure 8

Pairwise overlays of structures comparing the Rosetta models in each of the four states, highlighting the movements of the A' helix and the S4 helix.

a-d, Overlays of Rosetta structures as in Fig 6 (side view) between resting-apo versus resting-cAMP (a); resting-cAMP versus activated (b); activated versus inactivated (c); and resting-apo versus inactivated (d) states.

Electromechanical Coupling Mechanism for Activation and Inactivation of an HCN channel

Supplementary Table 1

Cβ-Cβ distances in the Rosetta models. between the adjacent, the same, and the diagonal subunits for the four FRET pairs

Distances (Å) between FRET Pairs	Resting-cAMP			Activated		
	Adjacent subunit	Same subunit	Diagonal subunit	Adjacent subunit	Same subunit	Diagonal subunit
W355-L481	21.3	40.3	28.6	14.6	34.1	27.3
W355-E506	18.1	34	36.5	13.5	35.7	35.3
W355-Y477	19.6	42.5	33.1	14.7	27.8	31.2
C369-Y477	19.2	27.7	30.5	23.4	28.8	35.1
Distances (Å) between FRET Pairs	Resting-apo			Inactivated		
	Adjacent subunit	Same subunit	Diagonal subunit	Adjacent subunit	Same subunit	Diagonal subunit
W355-L481	17.5	37.2	37	12.3	33	31
W355-E506	21.2	36.6	45.4	14.2	33.6	39.4
W355-Y477	17.7	30.5	39	13.5	26.5	34.2
C369-Y477	21	25.5	36.2	21.5	26.8	35.4

Electromechanical Coupling Mechanism for Activation and Inactivation of an HCN channel

Supplementary Table 2

Mutations for spHCN	Oligo Primers
L481C	*Forward:* 5'- GCA GGC AAT ACC GTG AGA AGT GCA AAC AAG TTG AAG AGT ACA TGC AG -3'<br>*Reverse:* 5'- CTG CAT GTA CTC TTC AAC TTG TTT GCA CTT CTC ACG GTA TTG CCT GC -3'
Y477C	*Forward:* 5'- CCA TGG ACT CCT CCA GCA GGC AAT GCC GTG AGA AGT TGA AAC AAG TTG AAG -3'<br>*Reverse:* 5'- CTT CAA CTT GTT TCA ACT TCT CAC GGC ATT GCC TGC TGG AGG AGT CCA TGG -3'
E506C	*Forward:* 5'- CGA TTA CTA CTG TTA CCG ATA CCG AGG AAA G -3'<br>*Reverse:* 5'- CGG TAA CAG TAG TAA TCG AGG ATC TTG TTT CG -3'
C369TAG	*Forward:* 5'- GCC TTC AAC GTA GCC AAT GCC GTC ATC CGG ATC TAG AAT CTA GTG GCT ATG ATG CTT CTG -3'<br>*Reverse:* 5'- CAG AAG CAT CAT AGC CAC TAG ATT CTA GAT CCG GAT GAC GGC ATT GGC TAC GTT GAA GGC -3'
W355TAG	*Forward:* 5'- CGG TTC GTC AGT CAA TAG GAA CAG GCC TTC AAC GTA GCC AAT G -3'<br>*Reverse:* 5'- C ATT GGC TAC GTT GAA GGC CTG TTC CTA TTG ACT GAC GAA CCG -3'
S346TAG	*Forward:* 5'- CTT CGA CTC CTG CGT CTG TAG AGG CTC ATG CGG TTC -3'<br>*Reverse: 5'-* GAA CCG CAT GAG CCT CTA CAG ACG CAG GAG TCG AAG -3'
L182H, L186H	*Forward: 5'-* GAC TGT CCT CCA ACC TTC AGA CAA TAG ACA CTC CAT GAA ACA TTT CGG AAG CAA GAA AGG -3'<br>*Reverse: 5'-* CCT TTC TTG CTT CCG AAA TGT TTC ATG GAG TGT CTA TTG TCT GAA GGT TGG AGG ACA GTC -3'

REFERENCES:

1 Dai, G. & Zagotta, W. N. Molecular mechanism of voltage-dependent potentiation of KCNH potassium channels. *Elife* **6**, doi:10.7554/eLife.26355 (2017).

2 Dai, G., Aman, T. K., DiMaio, F. & Zagotta, W. N. The HCN channel voltage sensor undergoes a large downward motion during hyperpolarization. *Nat Struct Mol Biol* **26**, 686-694, doi:10.1038/s41594-019-0259-1 (2019).
