# Supplementary material for: Electromechanical coupling mechanism for activation and inactivation of an HCN channel
Source: Nat Commun. 2021 May 14;12:2802. doi: 10.1038/s41467-021-23062-7 (PMC8121817; doi:10.1038/s41467-021-23062-7)
Supplement: Supplementary file 3 — Description of Additional Supplementary Files [file 41467_2021_23062_MOESM3_ESM.docx]

**Description of Additional Supplementary Files**

**Supplementary Movie 1: cAMP-induced conformational change of spHCN at 0 mV.**

Morph between the "resting-apo" and the "resting-cAMP" states, showing the conformational change of the spHCN channel due to cAMP binding at resting 0 mV. Left: Side view of the tetrameric spHCN channel; Right: bottom view of the same structure on the left. Each color represents a single subunit. cAMP is not shown.

**Supplementary Movie 2: cAMP-induced conformational change of spHCN at -100 mV.**

Morph between the "inactivated" and the "activated" states, showing the conformational change of the spHCN channel due to cAMP binding at hyperpolarized -100 mV. Left: Side view of the tetrameric spHCN channel; Right: bottom view of the same structure on the left. Each color represents a single subunit. cAMP is not shown.

**Supplementary Movie 3: Hyperpolarization-induced conformational change of spHCN in the presence of cAMP.**

Morph between the "resting-cAMP" and the "activated" states, showing the conformational change of the spHCN channel after applying -100 mV hyperpolarizing voltage in the presence of cAMP. Left: Side view of the tetrameric spHCN channel; Right: bottom view of the same structure on the left. Each color represents a single subunit. cAMP is not shown.

**Supplementary Movie 4: Hyperpolarization-induced conformational change of spHCN in the absence of cNMP.**

Morph between the "resting-apo" and the "inactivated" states, showing the conformational change of the spHCN channel after applying -100 mV hyperpolarizing voltage in the absence of cyclic nucleotide. Left: Side view of the tetrameric spHCN channel; Right: bottom view of the same structure on the left. Each color represents a single subunit.

**Supplementary Movie 5: Similarity in the A' helix rearrangement between the recovery of inactivation of spHCN channels and the activation of depolarization-activated KCNH channels**

Overlaying the morph (in cyan) between the "inactivated" and the "resting-apo" states for spHCN channels, with the morph (in orange) between EAG1 (PDB: 5K7L) and hERG1 (PDB: 5VA2), shown in a bottom view looking from the intracellular side.

**Supplementary Dataset 1: Rosetta model of “Resting-cAMP” state.**

**Supplementary Dataset 2: Rosetta model of “Activated” state.**

**Supplementary Dataset 3: Rosetta model of “Resting-apo” state.**

**Supplementary Dataset 4: Rosetta model of “Inactivated” state.**
