# Supplementary material for: Electromechanical coupling mechanism for activation and inactivation of an HCN channel
Source: Nat Commun. 2021 May 14;12:2802. doi: 10.1038/s41467-021-23062-7 (PMC8121817; doi:10.1038/s41467-021-23062-7)
Supplement: Supplementary file 9 — Reporting Summary [file 41467_2021_23062_MOESM9_ESM.pdf]

nature research

Corresponding author(s): William N. Zagotta and Gucan Dai

Last updated by author(s): March 21, 2021

Reporting Summary

Nature Research wishes to improve the reproducibility of the work that we publish. This form provides structure for consistency and transparency in reporting. For further information on Nature Research policies, see our Editorial Policies and the Editorial Policy Checklist.

Statistics

For all statistical analyses, confirm that the following items are present in the figure legend, table legend, main text, or Methods section.

n/a	Confirmed	
☐	☒	The exact sample size (n) for each experimental group/condition, given as a discrete number and unit of measurement
☐	☒	A statement on whether measurements were taken from distinct samples or whether the same sample was measured repeatedly
☐	☒	The statistical test(s) used AND whether they are one- or two-sided<br>*Only common tests should be described solely by name; describe more complex techniques in the Methods section.*
☒	☐	A description of all covariates tested
☒	☐	A description of any assumptions or corrections, such as tests of normality and adjustment for multiple comparisons
☐	☒	A full description of the statistical parameters including central tendency (e.g. means) or other basic estimates (e.g. regression coefficient) AND variation (e.g. standard deviation) or associated estimates of uncertainty (e.g. confidence intervals)
☐	☒	For null hypothesis testing, the test statistic (e.g. F, t, r) with confidence intervals, effect sizes, degrees of freedom and P value noted<br>*Give P values as exact values whenever suitable.*
☒	☐	For Bayesian analysis, information on the choice of priors and Markov chain Monte Carlo settings
☒	☐	For hierarchical and complex designs, identification of the appropriate level for tests and full reporting of outcomes
☒	☐	Estimates of effect sizes (e.g. Cohen's d, Pearson's r), indicating how they were calculated

Our web collection on statistics for biologists contains articles on many of the points above.

Software and code

Policy information about availability of computer code

Data collection	PatchMaster 2x73.2 (HEKA Elektronik), MetaMorph 7.8 (Molecular Devices)
Data analysis	RosettaCM 2020, Igor Pro 8, ImageJ 1.49, PyMOL 2.3.4

For manuscripts utilizing custom algorithms or software that are central to the research but not yet described in published literature, software must be made available to editors and reviewers. We strongly encourage code deposition in a community repository (e.g. GitHub). See the Nature Research guidelines for submitting code & software for further information.

Data

Policy information about availability of data

All manuscripts must include a data availability statement. This statement should provide the following information, where applicable:
- Accession codes, unique identifiers, or web links for publicly available datasets
- A list of figures that have associated raw data
- A description of any restrictions on data availability

Source data sets for Figs. 1, 2, 3, and 5 are included as Source Data files. Other data are available from the corresponding authors upon reasonable request.

Field-specific reporting

Life sciences study design

All studies must disclose on these points even when the disclosure is negative.

Sample size	Sample size was determined based on the conventions of patch-clamp experiments, and was similar to that in previous studies (e.g. Ref #15: Dai et al., NSMB, 2019).
Data exclusions	No data were excluded from analysis.
Replication	Experimental results were replicated 3-7 times from different membrane patches from different oocytes and different batches of oocytes.
Randomization	No randomization was used in this study. The experimental results were reproducible from day-to-day and were not dependent on any conditions such as the batch of oocytes, RNA, or solutions.
Blinding	The experiments and analysis were performed identically for the experimental and control constructs and were not prone to bias by the experimenter.

Reporting for specific materials, systems and methods

We require information from authors about some types of materials, experimental systems and methods used in many studies. Here, indicate whether each material, system or method listed is relevant to your study. If you are not sure if a list item applies to your research, read the appropriate section before selecting a response.

Materials & experimental systems

n/a	Involved in the study	
☒	☐	Antibodies
☒	☐	Eukaryotic cell lines
☒	☐	Palaeontology and archaeology
☐	☒	Animals and other organisms
☒	☐	Human research participants
☒	☐	Clinical data
☒	☐	Dual use research of concern

Methods

n/a	Involved in the study	
☒	☐	ChIP-seq
☒	☐	Flow cytometry
☒	☐	MRI-based neuroimaging

Animals and other organisms

Policy information about studies involving animals; ARRIVE guidelines recommended for reporting animal research

Laboratory animals	Xenopus laevis, female, approximately 3 years old.
Wild animals	This study did not involve wild animals.
Field-collected samples	This study did not involve samples collected from the field.
Ethics oversight	The animal-use protocols were consistent with the recommendations of the American Veterinary Medical Association and were approved by the Institutional Animal Care and Use Committee (IACUC) of the University of Washington.

Note that full information on the approval of the study protocol must also be provided in the manuscript.
